# Modelling Cerebrovascular Reactivity: A Novel Near-Infrared Biomarker of Cerebral Autoregulation?

**DOI:** 10.1007/978-1-4614-4989-8_13

**Published:** 2012-07-21

**Authors:** David Highton, Jasmina Panovska-Griffiths, Arnab Ghosh, Ilias Tachtsidis, Murad Banaji, Clare Elwell, Martin Smith

**Affiliations:** 1 Neurocritical Care, University College Hospitals, Queen Square, London, UK; 2 National Hospital for Neurology and Neurosurgery, Flat 40 Salisbury Mansions, St Anns Road, London, N153TP UK; 3 Medical Physics and Bioengineering, University College London, Malet Place, London, UK; 4 Institute of Neurology, University College London, Queen Square, London, UK; 5 Department of Mathematics, University of Portsmouth, Portsmouth, UK

**Keywords:** Modelling, Cerebrovascular reactivity

## Abstract

Understanding changes in cerebral oxygenation, haemodynamics and metabolism holds the key to individualised, optimised therapy after acute brain injury. Near-infrared spectroscopy (NIRS) offers the potential for non-invasive, continuous bedside measurement of surrogates for these processes. Interest has grown in applying this technique to interpret cerebrovascular pressure reactivity (CVPR), a surrogate of the brain’s ability to autoregulate blood flow. We describe a physiological model-based approach to NIRS interpretation which predicts autoregulatory efficiency from a model parameter *k_aut*. Data from three critically brain-injured patients exhibiting a change in CVPR were investigated. An optimal value for *k_aut* was determined to minimise the difference between measured and simulated outputs. Optimal values for *k_aut* appropriately tracked changes in CVPR under most circumstances. Further development of this technique could be used to track CVPR providing targets for individualised management of patients with altered vascular reactivity, minimising secondary neurological insults.

## Introduction

Cerebral blood flow (CBF) is tightly regulated by cerebral autoregulation (CA), forming a critical link between oxygen supply and demand. Myogenic, metabolic and neurological mechanisms lead to a complex pattern of vascular reactivity over different time scales combining to maintain constant perfusion across a wide range of perfusion pressure. Following brain injury, acute disturbances of CA may lead to hyper or hypo-perfusion and secondary neurological insults; maintaining cerebral perfusion is thus a core goal during the neurointensive care treatment of brain injury. However, delivering this is not straightforward as there is no convenient means of monitoring CBF or CA continuously at the bedside.

Measures of vascular reactivity, derived using surrogates of cerebral blood volume (CBV) or CBF, may be compared with arterial blood pressure (ABP) to investigate efficiency of cerebrovascular pressure reactivity (CVPR) and CA [1]. Recently near-infrared spectroscopy (NIRS) has been investigated in this regard as different NIRS indices reflect aspects of cerebral haemodynamics [2, 3]. Specifically, cerebral tissue oxygen saturation (TOS) and total haemoglobin have been applied as surrogates of CBF and CBV, respectively. When correlated with ABP these indices agree with well-established indices of CVPR [4, 5].

While these modes of analysis are simple and easily performed at the bedside, they do not account for the non-stationary and non-linear complexity within the range of measured signals. A model-based approach might make best use of the available data combining a priori knowledge of complex cerebral physiology with multiple measured variables to establish fully informed physiological predictions. This might account for additional important contributions to our interpretation of NIRS measured signals such as changes in CO_2_ or O_2_ tension, cerebral metabolic rate (CMRO_2_) and arterial to venous volume ratio.

We have previously described a physiological model of cerebral haemodynamics, oxygenation and metabolism and used this to aid interpretation of NIRS signals during cerebral physiological challenges in healthy volunteers [6]. The model combines haemodynamic, metabolic and oxygenation components creating simulated outputs of a range of measured signals. Variation in the model parameters from their basal values alters simulated outputs in a way which may mirror changes in underlying physiological processes. The model parameter *k_aut* has been designed to represent changes in the efficiency of CA ranging from 0 with an absence of CA to 1 where it is completely intact. This work translates our model [6] into the pathophysiological context of brain injury. The aim of this work is to use a range of measured signals, including NIRS, to identify a model-derived parameter as a biomarker of CA in individual patients.

## Methods

Three acutely brain-injured patients showing variation in CVPR were identified from an ongoing multimodal monitoring study in brain-injured patients. This study was approved by the institutional Research Ethics Committee and assent was gained from patient representatives.

For each patient dataset, CVPR was initially characterised using the pressure reactivity index (PRx) and mean velocity index (Mx) [1]. Two 30-min epochs were analysed for each patient, one with reactivity indices <0.3 suggesting intact CA and one >0.3 suggesting loss of CA. NIRS monitoring was performed with the NIRO 100 (Hammamatsu Photonics KK) ipsilateral to intraparenchymal intracranial pressure (ICP) monitoring and transcranial Doppler flow velocity of the middle cerebral artery (Vmca) (DWL Doppler Box, Compumedics, Germany). NIRS measurements included spatially resolved tissue oxygenation index (TOI) and normalised total haemoglobin index (nTHI) representing measures of TOS and total haemoglobin, respectively. Changes in concentration of oxyhaemoglobin (∆[HbO_2_]) and deoxyhaemoglobin (∆[HHb]) were determined by the modified Beer–Lambert method. Invasive ABP from a radial artery catheter, end tidal CO_2_ (ETCO_2_) and pulse oximetry (SpO_2_) were gathered through an Intellivue monitor (Philips, N.V., Amsterdam, The Netherlands). Signals were synchronised, downsampled to 1 Hz and filtered with a lowpass 0.1 Hz fifth-order Butterworth filter to remove high frequency noise and respiratory influences. Of these measured signals ABP, ETCO_2_ (approximating PaCO_2_), SpO_2_ and ICP were used as model inputs. These produced simulated outputs for CBF, total haemoglobin ([HbT]), [HbO_2_], [HHb] and TOS which were compared with their measured counterparts Vmca and NIRS (nTHI, ∆[HbO_2_], ∆[HHb], TOI).

Optimisation was performed by minimising the difference between measured signals and simulated outputs for Vmca and NIRS finding optimal values for parameter *k_aut* (representing CA) and an additional parameter *u* reflecting cerebral energy demand. Reduction of this additional parameter below its basal level simulating a reduction in cerebral metabolism was required to adequately fit the measured NIRS signals. This seems physiologically plausible because all patients were deeply sedated at the time of study. *k_aut* values produced by these different optimisation strategies were compared to index-based predictions of CVPR for consistency. The difference between simulated outputs and measured signals is expressed as the mean absolute difference between the two. The improvement in the fit following optimisation is given as the percentage difference between measured signals and simulated outputs at basal parameter settings and optimised parameter settings divided by the basal value.

## Results

**Fig. 13.1:**
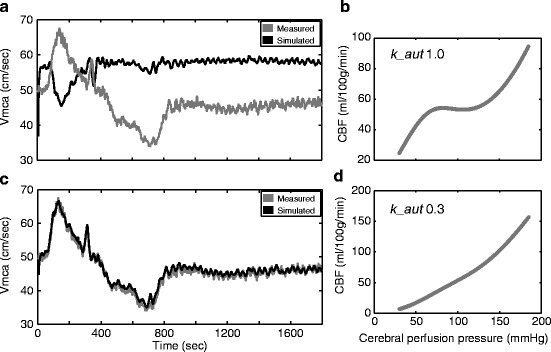
Measured signals and simulated outputs for a patient with low CVPR. (**a**) Measured Vmca and simulated Vmca using the basal *k_aut* value (1.0). (**b**) The steady-state relationship between CPP and CBF using the basal value of *k_aut* reproduces a typical normal static autoregulation curve. (**c**) Measured and simulated Vmca post-optimisation of *k_aut* demonstrate excellent agreement compared with the basal value. This value of *k_aut* (0.3) is low and in the dysautoregulated range suggesting that a loss of CA is required to explain the measured signals. (**d**) The predicted steady state between CPP and CBF using the optimised value for *k_aut* (0.3). This closely resembles a static autoregulation curve with loss of CA

A high *k_aut* was associated with intact CVPR and a low value disturbed CVPR in all simulations excluding those optimised on the basis of TOI. An example dataset is shown in Fig. 13.1 demonstrating disturbed CA. It can be seen that simulation with a low value of *k_aut* (0.3), reflecting dramatically impaired autoregulation, simulates Vmca and nTHI most accurately.

Model simulations using different measured signals for optimisation of *k_aut* varied in the relationship between *k_aut* and predicted CVPR. When *k_aut* is optimised by minimising the difference between measured Vmca and simulated CBF alone in all epochs (Table 13.1), there is accurate prediction of Vmca (mean absolute difference 1.98 cm/s). Post-optimisation *k_aut* values are lower in the epochs with reduced CVPR (0.53), suggesting that *k_aut* appropriately reflects the level of CA. When measured NIRS signals are included in this strategy (Table 13.2) it is possible to account for the changes in nTHI by optimising *k_aut* alone. This continues to predict appropriate values of *k_aut* (Table 13.2, column 1). To adequately fit measured and simulated ∆[HbO_2_] and ∆[HHb], optimisation of *u* reducing cerebral metabolism was required. Again, this approach predicts lower values of *k_aut* (0.47) in those with reduced CVPR. However, to achieve the best fit requires a value for *u* that is unphysiologically low.

**Table 13.1:** Optimisation of *k_aut* using simulated CBF against measured Vmca alone

	Improvement (%)	Mean absolute difference	Optimal *k_aut*	
Vmca	59 (35)	1.97 (0.78) cm/s	CVPR intact	0.87 (0.12)
nTHI	28 (25)	0.009 (0.01) au	CVPR lost	0.53 (0.25)
∆[HbO_2_]	−18 (36)	1.86 (2.5) μmol/L		
∆[HHb]	−52 (65)	1.58 (2.2) μmol/L		
TOI	−70 (75)	17 (2) %		

Mean (SD) improvement between basal *k_aut* and optimised *k_aut* show improved prediction of Vmca and nTHI. Mean (SD) absolute differences between measured signals and simulated outputs are shown demonstrating accurate prediction of Vmca and nTHI. Post-optimisation *k_aut* values appropriately reflect the measured CVPR with a lower mean *k_aut* where CVPR is lost

**Table 13.2:** Optimisation of *k_aut* and *u* based on different combinations of measured signals

Measured signals used to optimise against	Vmca; nTHI	Vmca; nTHI; ∆[HbO_2_]; ∆[HHb]	Vmca; nTHI; ∆[HbO_2_]; ∆[HHb]; TOI
*Optimal k_aut value*			
CVPR intact	0.93 (0.17)	0.83 (0.06)	0.03 (0.52)
CVPR lost	0.37 (0.15)	0.47 (0.55)	0.9 (0.69)
Optimal *u* value	0.50 (0.55)	0.00 (0.00)	0.67 (0.52)
*Improvement*			
Vmca	70 (28) %	70 (26) %	50 (31) %
nTHI	36 (30) %	36 (31) %	5 (34) %
∆[HbO_2_]	14 (48) %	49 (26) %	−40 (74) %
∆[HHb]	−6 (82) %	78 (16) %	−26 (106) %
TOI	−536 (894) %	−633 (1,113) %	14 (57) %

For each column different measured signals were compared to model outputs to find optimal values for *k_aut* and *u*. Mean (SD) improvement between basal parameter values and optimised values are shown demonstrating improved post-optimisation prediction of measured signals (excluding TOI). Optimal *k_aut* values for each optimisation strategy are shown and are consistent with levels of measured CVPR except where TOI is included in the optimisation

**Fig. 13.2:**
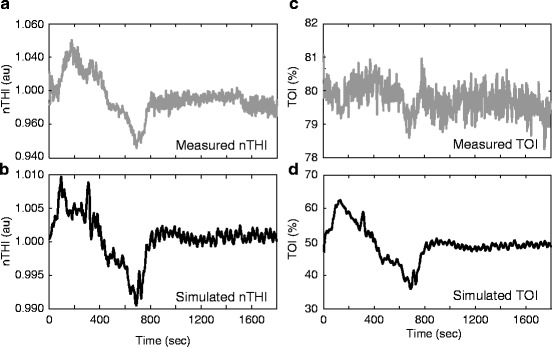
Measured and simulated NIRS outputs from dataset in Fig. 13.1. (**a**) Measured nTHI and (**b**) simulated nTHI demonstrate moderate agreement. (**c**) Measured TOI and (**d**) simulated TOI agree qualitatively only

Inclusion of TOI within the optimisation strategy is problematic and it is not possible to fit TOI well in combination with other measured signals (Table 13.2, column 3). Optimal values for *k_aut* do not reflect the level of predicted CA in this final approach. The behaviour of measured TOI differs significantly from simulated outputs of TOS; large simulated changes in TOS result from large changes in CBF which are not present in the measured TOI (Fig. 13.2).

## Discussion

We have identified a model parameter *k_aut* which simulates changes in CA and improves prediction of NIRS signals in brain injury. The optimal value of *k_aut* may thus represent a composite biomarker of cerebral autoregulatory function informed from multiple NIRS inputs. This approach aims to form cohesive physiological predictions based on prior knowledge of physiology, maximising the potential of the available data.

Fitting nTHI was least problematic probably because fewer physiological processes influence this signal. In comparison ∆[HbO_2_], ∆[HHb] and TOS encode metabolic components and differential effects of arterial and venous components become more influential. It was impossible to achieve an adequate fit for TOS by varying only the model parameters *k_aut* and *u*. Despite qualitative agreement, the magnitude of variation and baseline saturation showed large discrepancies. It is unlikely that further optimisation within physiological plausibility could explain the lack of variability despite the large changes in CBF observed. However, a differing baseline is more easily explained. Similar observations were made during studies in healthy volunteers [7], but in this case TOS could be explained by adjusting the extracerebral:intracerebral signal weighting to 80:20 or doubling the venous volume, both of which seem unlikely. Studies such as these indicate that accurate prediction and interpretation of TOS might require combined modelling of cerebral physiology and light transport in tissue.

This study was of limited power including only six epochs from three patients. However these datasets demonstrate an extreme of physiological dysfunction with large changes in ABP and CBF, representing an excellent challenge for our model. Further work must include large numbers of patients undergoing a range of physiological challenges to increase the quality of measured signals in patients with lesser degrees of impaired CA. Although this approach has not necessarily been followed for many established indices of CVPR it should be viewed as a prerequisite to translation into the clinic.

With further investigation, model-informed interpretation of NIRS signals might offer enhanced prediction of CA across widely varying physiological and pathophysiological contexts. Prior knowledge of population characteristics and further model simplification should improve computational efficiency and move toward bedside implementation. This form of interpretation progresses beyond simple correlation analyses by combining information from multiple NIRS and systemic measures with a priori knowledge of physiology to provide cohesive predictions of cerebral well-being. Thus, use of *k_aut* as a biomarker of CA efficiency could inform pathophysiology and potentially provide a target for physiological optimisations to improve outcome.
